# Hepatitis A: A Case Report Example of a Growing Epidemiological Threat

**DOI:** 10.51894/001c.7436

**Published:** 2019-03-04

**Authors:** Adam Foster, Stephanie Hernandez

**Affiliations:** 1 Emergency Medicine McLaren Macomb

**Keywords:** public health, epidemiology, jaundice, hepatitis a

## Abstract

Hepatitis A is a common worldwide cause of acute hepatitis. It has been classically associated with epidemics and is increasingly prevalent in the developing world. Generally, the illness is self-limited and only requires supportive management, reassurance, and proper hygiene instructions. This case involves a male in his early 30s who presented non-emergently with jaundice and a weeklong history of fatigue, nausea, and flu-like symptoms. The patient underwent laboratory and radiological evaluation. Test results revealed a significant transaminitis, hyperbilirubinemia, and suggestion of cholecystitis. Further testing did reveal hepatitis A infection. This case illustrates the importance of clinicians having a high clinical suspicion for the disease based on individual risk factors as this disease can have a profound epidemiological impact in terms of local outbreaks and public health expenses.

## INTRODUCTION

Hepatitis is a generic term that refers to some manner of liver inflammation. The most common causes leading to such a diagnosis include viral infections and chronic alcohol abuse.^1^ Other causes can include bacterial, fungal, parasitic, immunologic, and toxic exposures. Hepatitis A Virus (HAV) is a virus that is spread almost exclusively through the fecal-oral route, although there does exist a very rare ability for blood transmission.^1^ HAV is an acute illness and there is no associated chronic carrier state (i.e., asymptomatic person capable of transmitting) such as seen with Hepatitis B Virus (HBV) or Hepatitis C Virus (HCV). Typically, cases occur in association with epidemics, as opposed to sporadic cases, with the most common risk factor for transmission being travel outside of the US.^1^

The incubation period for HAV ranges from 14 to 45 days, with a relatively short duration of viremia (i.e., detectable virus in the blood) and maximum infectivity to others that is most prominent before symptom onset.^1^ Differentiation of the cause of hepatitis generally requires a broad laboratory evaluation and thorough history and physical exam.

Although HAV is rarely diagnosed initially in the Emergency Department (ED) due to serology testing times,^1^ a high clinical suspicion for the disease can lead to timely intervention including contact precautions and prevention of complications. Primary and secondary prophylaxis is available, however vaccination is not mandatory as the disease is rarely fatal, has no chronic carrier state, and has an overall low incidence in the United States.^2^

## METHODS

### Case Report

A Caucasian male in his early 30s presented to the ED with the chief complaint of yellowing of his skin and typically white sclera of his eyes. This was preceded by five days of progressive fatigue and flu-like symptoms. He also admitted to having produced several tan-colored bowel movements, dark urine, subjective fevers, and nausea. He denied any abdominal pain, vomiting, diarrhea, hematuria, or rashes. He also denied having completed any recent travel, insect or chemical exposures, or any known sick contacts.

He did not recall any peculiar food exposures during the prior week. He denied ever having experienced symptoms like this in the past but did admit to current HIV prophylaxis medication for the reason of “being smart.” He did admit to previous intravenous drug use with last administration three years prior. He had a history of mild well-controlled asthma and denied any previous surgeries.

The patient’s immediate vitals revealed hemodynamic stability with heart rate of 106, respiratory rate of 18, temperature of 97.6 degrees axillary, blood pressure of 140/86, and oxygen saturation of 100% on room air. His physical exam revealed a well-nourished, diffusely jaundiced male in no acute distress. The patient was alert and oriented and answering all questions appropriately, albeit with short answers.

Further examination revealed prominent bilateral yellow discoloration of the eyes (i.e., scleral icterus) and abdominal examination demonstrated a mildly distended abdomen with mild tenderness in the right upper quadrant. There were no other indications of peritonitis and the remainder of the physical examination was within normal limits. The patient was provided with intravenous (IV) fluids and a broad laboratory evaluation and computerized axial tomography (CT) of the abdomen and pelvis with IV contrast was obtained.

CT of the abdomen and pelvis was interpreted as gall bladder contraction with wall edema and mucosal hyper-enhancement. (Figure 1) No gallstones were identified and the liver, common bile duct and pancreas were all within normal limits. Laboratory evaluation was obtained to evaluate for the degree of liver impairment and was pertinent for thrombocytopenia (88,000), hyperglycemia (451 mg/dL), hyperbilirubinemia (9.2 mg/dL), transaminitis (2238 U/L and 3806 U/L), and elevated PT-INR (i.e., prothrombin time-international normalized ratio) of 15.9 sec/1.54. (Table 1). These laboratory abnormalities suggested possible new-onset Diabetes Mellitus as well as significant liver dysfunction.

**Figure:**
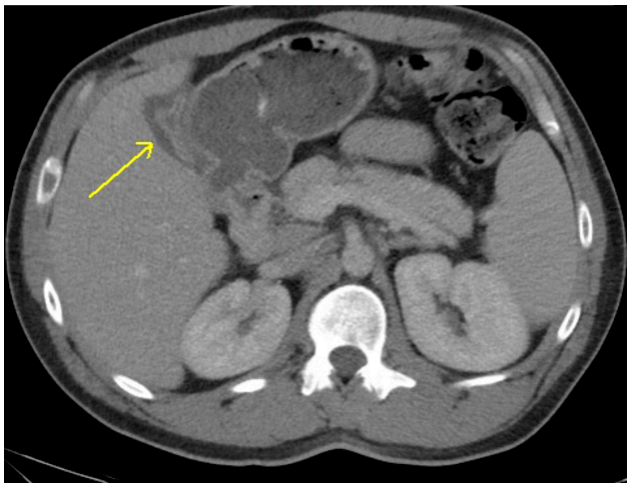
Figure 1 CT Abdomen/Pelvis Axial Slice Demonstrating Gall Bladder Contraction with Wall Thickening

**Table:** Table 1 Emergency Department Laboratory Evaluation

**White Blood Cell**	4.09 x10^3^ per µL (normal range 4.0-10)	**Sodium**	132 mEq/L
			(135-145)
**Hemoglobin**	16.3 g/dL	**Potassium**	3.8 mEq/L
	(13-17)		(3.5-5)
**Platelets**	88 x10^3^ per µL (150-400)	**Chloride**	95 mEq/L
			(95-105)
		**Carbon Dioxide**	25 mEq/L
			(20-29)
**HIV, Rapid**	Non-reactive	**Blood Urea Nitrogen**	15 mg/dL
			(8-21)
**Influenza A/B**	Negative	**Glucose**	451 mg/dL
			(65-110)
		**Creatinine**	0.990 mg/dL
			(0.8-1.3)
**Alkaline Phosphatase**	484 U/L	**Prothrombin/INR**	15.9 sec/1.54
	(50-100)		(11-14)/(0.9-1.2)
**Bilirubin, total**	9.20 mg/dL	**Calcium**	8.00 mg/dL
	(0.1-1.2)		(8.5-10.2)
**Bilirubin, direct**	7.78 mg/dL	**Lactic Acid**	1.4 mmol/L
	(<0.3)		(0.5-2.0)
**Bilirubin, indirect**	1.42 mg/dL		
	(<0.7)		
**Aspartate Transaminase**	2238 U/L		
	(5-30)		
**Alanine Transaminase**	3806 U/L		
	(5-30)		
**Lipase**	193 U/L		
	(10-150)		

Given the radiological findings and suspicion for obstructive jaundice with possible gall bladder infection (i.e., cholecystitis), the authors discussed the case with general surgery. Orders for a magnetic resonance cholangiopancreatography (MRCP) and a viral hepatitis panel were placed for suspected concomitant acute hepatitis. The patient was initiated on IV antibiotics for suspected cholecystitis.

The case was then discussed with the Gastroenterology (GI) service who had also recommended a MRCP, avoidance of hepatotoxic medications (e.g., acetaminophen and ciprofloxacin) and hepatitis panel. The patient was subsequently admitted to the hospital in hemodynamically stable status with MRCP and hepatitis panel pending. Gastrointestinal (GI), general surgery and endocrinology for suspected new-onset Diabetes Mellitus were consulted on the case.

During the patient’s two-day hospital course, he underwent a MRCP which showed no gallstones (i.e., cholelithiasis), intra or extra-hepatic biliary dilatation, choledocholithiasis, or pancreatic ductal dilatation. The patient’s newly diagnosed Diabetes Mellitus (Hemoglobin A1c of 9.2) was managed by Endocrinology. A hepatitis panel indicated no evidence of HBV or HCV reactivity but was reactive for anti-HAV immunoglobulin (IgM), suggesting acute HAV.

The patient was again evaluated by the same consulting services with further recommendations of no surgical intervention being required and trending of the hepatic function panel. On the patient’s hospital Day 2, his repeat hepatic function lab panel revealed improvement in the patient’s liver enzymes and function as well as his PT-INR. The patient was subsequently discharged after receiving medical clearance from the consultants with strict clinic-based follow-up. This entailed appropriate counseling with regards to risk factor modification as well as ensuring resolution of jaundice and viral shedding.

## DISCUSSION

Patients affected by HAV can have a highly variable clinical presentation ranging from asymptomatic to fulminant (i.e., a severe sudden onset) liver failure.^1^ A significant number of those patients affected are actually asymptomatic, but malaise, fever, and anorexia are the most common presenting symptoms if they occur.^1^ These vague symptoms are generally followed by nausea, vomiting, diarrhea, abdominal discomfort, and the eventual development of jaundice.^3^ Fulminant HAV, on the other hand, is exceedingly rare occurring in only 1-2% of cases.^4^ This is characterized by hepatic failure and progressive encephalopathy (i.e., brain pathology) over a period of days.^4^

Patients may also present with rare and specific symptoms as in this case including pale (i.e., acholic) stools and dark urine, both of which are indicative of a conjugated hyperbilirubinemia. This suggests an inability of the liver to expel bilirubin from the bile ducts, either from intrinsic hepatocyte dysfunction or an external obstruction or both. This is compared to an unconjugated hyperbilirubinemia that would typically suggest an abundance of bilirubin being produced for a myriad of reasons.^3^ Physical examination findings typically include scleral and/or cutaneous icterus, abdominal tenderness and palpable hepatomegaly.^3^ Aside from fever, other vital sign abnormalities may be present, especially with concomitant vomiting, such as orthostatic hypotension and tachycardia.

When managing a patient presenting with jaundice and suspicion for hepatitis, it is especially important to gather a thorough history from the patient and their recent contacts if possible. This history should include any known sick contacts, travel history, illicit drug use, animal exposures, family history, or similar occurrences in the past. Any of these risk categories should lead clinicians to suspect some form of hepatitis as a cause of the patients’ presenting symptoms.

Clinicians being aware of current local epidemiological trends can also be of diagnostic benefit. As of the time of this writing, there had been a dramatic rise in the number of reported cases of acute HAV in Southeastern Michigan. The Michigan Department of Health and Human Services had reported that as of 3/21/18 (beginning 8/1/16) there have been 789 reported cases of HAV related to the outbreak.^5^ This condition contributed to 635 hospitalizations (80.5%) and 25 deaths (3.2%).

Of note, Macomb County has the highest number of reported cases at 212 cases which is more than the city of Detroit (i.e, 166 cases).^5^ The outbreak is believed to be linked to county-wide opioid and heroin use patterns as over half of the reported cases has some connection with this factor. As in this case, our patient admitted to a history of IV drug use and as was later determined during his hospitalization he also admitted to engaging in other high-risk behaviors including sex with other men.^6^

The economic impact of HAV outbreaks has been reviewed both globally as well as on a national level. One 2003 study in particular looked at a Spokane, Washington outbreak and estimated each case of HAV cost $2,683.^7^ Most of the expenditures were associated with hospital admissions and lost productivity in the community was also a major indirect factor. The expense of these endemics when compared to vaccination programs and other preventative public health initiatives continues to be an area of epidemiological interest.^8^

A HAV diagnosis is typically not made in the ED but suspected cases can be managed expectantly (i.e., monitored closely before treatment) while definitive studies are pending. The differential diagnosis includes bacterial, viral, fungal, parasitic, and alcoholic hepatitis.^1^ Also included are causes of extra-hepatic obstruction such as cholelithiasis, cholecystitis, choledocholithiasis, and malignancy of the biliary and pancreatic tissue.^1^ Diagnostic imaging is usually indicated in the form of a right upper quadrant ultrasound and possible CT of the abdomen and pelvis.^3^

More advanced imaging may be indicated in the form of a MRCP to further distinguish biliary pathology and possibly intervene on a cause of obstruction. The most critical laboratory studies to obtain include a hepatic function panel to assess degree of liver enzyme elevation (transaminitis), hyperbilirubinemia, and PT-INR which serves as the most accurate representation of hepatic impairment.^4^ Definitive studies for acute HAV (as well as HBV and HCV) can be obtained through a viral hepatitis panel. Acute HAV is indicated by positive Anti-HAV IgM whereas IgG indicates past exposure (when not co-existing with IgM).^9^

Management of acute HAV includes IV fluids and electrolyte correction, anti-emetics, and avoidance of hepatotoxic medications (e.g., acetaminophen and ciprofloxacin) and alcohol intake.^1^ Antiviral and antibiotic medications are not indicated in uncomplicated acute HAV.^1^ Hospitalization is generally reserved for those with intractable vomiting, severe electrolyte or fluid imbalance, altered mental status, a PT-INR greater than 1.5, or any other evidence of fulminant disease.^1^ Otherwise stable individuals can be safely discharged with a presumptive diagnosis and strict clinic-based gastroenterology follow-up. Patients should be instructed on strict hand hygiene and those working in the food industry should delay return to work until their jaundice has resolved.^6^

According to the latest federal Centers for Disease Control (CDC) guidelines, unvaccinated persons who have been exposed recently to HAV should be administered one dose of the single-antigen HAV vaccine or immune globulin (IG) as soon as possible and within two weeks after exposure.^9^ IG is preferred for those less than 12 months old and greater than 40 years old, immunocompromised persons with chronic liver disease, and those who are allergic to the vaccine.^9^

As for primary prophylaxis, the CDC and the Advisory Committee on Immunization Practices (ACIP) recommends that all children at one year of age, those at increased risk for infection or complications from HAV, and any person wishing to obtain immunity should receive the vaccination.^6^ The HAV vaccine has been available since 1995 and has resulted in a 95% decline in the incidence of disease.^6^ Despite the apparent success of the vaccine, mandatory administration is not the norm owing largely to the fact that the disease is rarely fatal, has no chronic carrier state, and has an overall low incidence in the United States.^2^

## CONCLUSIONS

In this paper, the authors reported on the presentation of a patient with an acute HAV infection. A presumptive diagnosis of hepatitis was made in the ED based on the patient’s historical risk factors with symptomatology, coupled with supporting laboratory findings. The case was complicated by the findings on imaging suggestive of acute cholecystitis. Appropriate lab value serologies were obtained and supportive care was provided which resulted in a short hospital admission with gradual improvement in symptoms and liver function. This case illustrates the importance of clinicians observing a broader differential diagnosis as well as having an understanding of the illness course and possible complications. This case report example further stresses the significance of clinicians considering local epidemiological trends and how this may aid in diagnosis and appropriate management thereafter.

### Conflict of Interest

The authors declare no conflict of interest.
